# Molecular Classification of Hepatocellular Adenomas

**DOI:** 10.1155/2013/315947

**Published:** 2013-01-15

**Authors:** Jean Charles Nault, Jessica Zucman Rossi

**Affiliations:** ^1^Inserm UMR-674, Génomique Fonctionnelle des Tumeurs Solides, IUH, 75010 Paris, France; ^2^Labex Immuno-Oncology, Faculté de Médecine, Université Paris Descartes, Sorbonne Paris Cité, 75005 Paris, France; ^3^Hopital Europeen Georges Pompidou, Assistance Publique-Hôpitaux de Paris, 75015 Paris, France

## Abstract

Hepatocellular adenomas (HCAs) are benign tumors developed in normal liver most frequently in women before menopause. HCAs lead to diagnostic pitfalls and several difficulties to assess the risk of malignant transformation in these young patients. Recent advances in basic knowledge have revealed a molecular classification related to risk factors, pathological features, and risk of transformation in hepatocellular carcinoma. Three major molecular pathways have been identified altered in specific HCA subgroups that are defined by either (1) inactivation of hepatocyte nuclear factor 1A (*HNF1A*) transcription factor, (2) activation of the WNT/**β**-catenin by *CTNNB1* mutations, or (3) activation of the IL6/STAT3 pathway by somatic mutation of *IL6ST*, *GNAS*, or *STAT3*. Here, we will review the different molecular classes of HCA.

## 1. Introduction

Hepatocellular tumors deriving from monoclonal proliferation of hepatocytes are classically divided in benign hepatocellular adenoma (HCA) and malignant hepatocellular carcinoma (HCC). HCAs are rare tumors most frequently-developed in women before menoaupose and after a long-term use of oral contraception [1]. Other risk factors such as glycogen storage diseases and androgen intake are also classically associated with HCA development. HCA could be complicated frequently by hemorrhage and more rarely by malignant transformation in HCC [2, 3]. For a long time, HCA was considered as a benign monoclonal proliferation of hepatocytes driven by oestrogen exposition [4, 5]. However, molecular classification has redrawn the physiopathological and clinical landscape of HCA [6]. This new classification linked specific risk factor, clinical history, and histological features to each molecular subgroup of HCA [6–9]. In addition, this genotype/phenotype classification has been validated by several groups worldwide demonstrating its robustness and its wide reproductibility in clinical practice [10–15]. In this paper we aimed to describe how genomic analyses enabled us to identify the different HCA molecular subgroups and their specific molecular defects. 

## 2. Molecular Classification of Hepatocellular Adenomas

### 2.1. Adenomas Inactivated for *HNF1A* (H-HCA)

In 2002, we identified *HNF1A*, as the first driver gene inactivated by mutation in hepatocellular adenomas [16]. *HNF1A* codes for the hepatocyte nuclear factor  1  A, a transcription factor involved in hepatocyte differentiation and metabolism control [17]. Previously, in 1996, Yamagata and collaborators had identified germline mutations of *HNF1A* as the causal alteration of the specific diabetes named MODY 3 for maturity onset diabetes of the young type 3. In MODY3 patients, one allele of *HNF1A* is inactivated in all cells of the organism showing the pivotal role of *HNF1A* partial inactivation in glucose homeostasis dysregulation [18]. In HCA tumor cells, we described complete *HNF1A* inactivation by mutation of both alleles in 35% to 45% of the cases (Table 1) [16]. In most of the cases, both mutations occurred in tumor cells and were of somatic origin. However, in 10% of HCA inactivated for *HNF1A*, one mutation was germline, and consequently, we identified MODY3 patients developing HCA [19, 20]. These patients could also have adenomatosis, a rare condition defined of more than 10 adenomas in the liver [21, 22]. In this line, these results have revealed for the first time *HNF1A* as a tumor suppressor gene in addition to its role in metabolism regulation. We further showed that *HNF1A* inactivation induces in hepatocyte dramatic alteration in metabolic pathways and epithelial-mesenchymal transition that can participate to tumor development [23, 24]. 

In addition the of environmental factor and germline *HNF1A*-mutations, others genetic features could predispose to the occurrence of *HNF1A* mutated adenomas. In this line, we identified heterozygous germline mutations of *CYP1B1* in a subset of patients with H-HCA [25]. All patients with these mutations have a decrease enzymatic activity of the cytochrome p450 *CYP1B1*. Because CYP1B1 is involved in the metabolism of estrogens, it suggests that development of H-HCA could be promoted by a defect in this pathway in relation with exposure to oral contraception.

At the pathological level, HCA with *HNF1A* biallelic mutations exhibited typical features. They are characterized by diffuse steatosis in tumor hepatocytes [6]. We further showed that the homogeneous accumulation of lipids in tumor hepatocytes was related to an increase of fatty acid synthesis induced by *HNF1A* inactivation [26]. H-HCA can be easily diagnosed using pathological examination because these adenomas are characterized by a constant and specific lack of FABP1 expression in the tumor hepatocytes [12, 27].

### 2.2. *β*-Catenin Activated Adenomas (*β*-HCA)

The Wnt/catenin pathway is a pivotal oncogenic pathway involved in solid and haematopoietic tumors. *CTNNB1,* the gene coding for *β*-catenin, is activated by somatic mutation in a large number of different tumor types like medulloblastoma or breast cancer [28]. Moreover, it is the most frequently mutated oncogene in hepatocellular carcinoma (from 20 to 40% of the cases) [29]. *CTNNB1*-activating mutations target few serine and threonine amino acids in *β*-catenin, residues that are physiologically phosphorylated by the APC/GSK3/axin complex inducing degradation of *β*-catenin by the proteasome. *CTNNB1* mutations impaired the phosphorylation by the APC/GSK3B/AXIN complex and led to the translocation of *β*-catenin into the nucleus [28, 30]. In this condition, the oncogenic effect of *β*-catenin is fully active [31, 32].

Mutations activating *β*-catenin are described in 10 to 15% of HCA (Table 1) [6, 33]. Male are overrepresented in this subgroup of HCA [34]. Furthermore, *β*-HCA are often characterized by pseudoglandular formation, cell atypia, and cholestasis at the pathological level. Using immunochemistry, we showed that *β*-HCAs are characterized by a strong cytoplasmic expression of glutamine synthase and nuclear expression of *β*-catenin in tumor hepatocytes. However, despite a good specificity, these markers have a lack of sensitivity for the diagnosis of *β*-HCAs and HCA should be screened for *CTNNB1* mutations [12, 27, 35, 36], when glutamine synthase and *β*-catenin markers are not informative.

Importantly, we showed that HCA with activating mutations of *β*-catenin have a high risk of malignant transformation in HCC [6, 36, 37]. Moreover, distinguishing HCA from well-differentiated HCC developed on normal liver could be challenging. Consequently, all HCA harboring a mutation of *β*-catenin should be surgically resected in order to avoid the risk of malignant transformation. In this context, the performance of immunohistochemical marker developed to discriminate high-grade dysplastic nodules from very early HCC (like glutamine synthase, glypican 3 or hsp70) on cirrhosis remains poorly explored to differentiate HCA from very well differenciated HCC on normal liver and should be used with caution [38]. A recent study has shown that the combination of glypican 3 and HSP70 has a good specificity (100%) but an insufficient sensitivity (43%) to distinguish HCA from well-differenciated HCC [38, 39]. However, the small numbers of tumors analyzed preclude the generalization of these markers in clinical practice and required additional studies. Another concept is that some hepatocellular tumors will remain borderline tumors between HCA and HCC despite histological analysis by an expert pathologist. In this grey zone, *CTNNB1* mutations are also overrepresented [6, 34].

In this line, screening for *CTNNB1* mutation should be mandatory to detect HCA with a potent risk of malignant transformation and borderline lesion between HCA and HCC that should be resected. 

### 2.3. Inflammatory Adenomas (IHCAs)

In the physiological point of view, the most important breakthrough has been performed by the identification of the so-called “inflammatory HCA” and dissection of  IL6/JAK/STAT pathway [40, 41].

IHCAs are characterized by the activation of JAK/STAT and interferon I and II pathway [40, 42]. This subtype of adenomas exhibited strong pathological hallmark: inflammatory infiltrates, dystrophic arteries, and sinusoidal dilatation [43]. Immunohistochemical marker could be used as diagnostic tool for this subtype of HCA. Inflammatory HCA exhibited a cytoplasmic overexpression of SAA and CRP, two proteins of the acute phase of inflammation, in the tumor hepatocytes (Table 1) [12, 15]. Sometimes, IHCAs are associated with inflammatory syndrome and related anemia [44]. Peripheral inflammatory syndrome can regress after resection of the tumor, and it could be considered as a “paraneoplastic syndrome” [45, 46]. IHCA occurred more frequently in patients with high alcohol consumption and obesity, two conditions associated with chronic cytokine production [6, 46]. We also described an IHCA transformed in HCC mutated for both gp130 (*IL6ST*) and *β*-catenin (*CTNNB1*) and developed on the background of Castleman disease [47]. In this rare disease, a chronic IL6 systemic secretion is produced by a lymphoproliferative disorder. It underlined again the possible role of chronic cytokine production (obesity, high alcohol consumption, and Castleman disease) as a predisposing factor to inflammatory HCA occurrence. Recently, we deciphered the molecular alterations leading to the activation of inflammatory pathway in the tumor hepatocytes. 

We described the oncogenes that explain the hepatocytes proliferation and the inflammatory phenotype (“oncogene-induced inflammation”). The most preeminent oncogene identified was gp130 (*IL6ST*) [42]. 65% of inflammatory HCAs exhibit a somatic activating mutation of gp130. Gp130 is the coreceptor of IL6R. Activating mutations of gp130 led to the constitutive activation of the JAK/STAT pathway in the absence of the IL6 ligand [42, 48]. A small subset of HCC exhibited both gp130 and *β*-catenin-activating mutations. Interestingly, these HCC are developed in normal liver and could be derived from HCA. 

We also described for the first time in human tumors somatic mutations activating *STAT3 *[49]. These mutations explained the uncontrolled activation of JAK/STAT pathway and the observed phenotype in 6% of the IHCA. Finally, we discovered *GNAS*-activating mutations in 5% of inflammatory HCA [50]. *GNAS* gene coded for alpha subunit of Gs protein and is a well-known oncogene in pituitary and thyroid adenomas. Mutations of *GNAS* gene impaired the GTPase activity of alpha subunit and led to its permanent activation by an unregulated binding of GTP. As a consequence, cyclic Amp accumulates in the cells [51]. In adenoma, we described a crosstalk between cyclic Amp and JAK/STAT pathway that explained the mild inflammatory phenotype in *GNAS*-mutated HCA [50]. In this line, we also described HCA in patients with McCune Albright syndrome [52]. McCune-Albright syndrome is an orphan disease due to somatic postzygotic mosaic *GNAS* mutation. This genetic disorder is characterized by pituitary and thyroid adenomas, fibrous bone dysplasia, and “café au lait” skin macula [51]. Consequently, McCune Albright syndrome also predisposed to HCA development.

### 2.4. Unclassified Adenomas

Finally, 10% of HCAs have no known genetic alterations or specific histological phenotype (Table 1) [34]. The molecular drivers of this subtype of HCA remain to be determined.

## 3. Mechanism of Development of  Hepatocellular Adenomas: A Contribution of Different Genes with a Genotoxic Signature

In the canonical point of view, malignant hepatocellular tumors (HCC) arise on chronic liver disease, mainly cirrhosis or chronic HBV infection, whereas hepatocellular benign tumors are developed on normal liver. However, several clinical, pathological, and molecular observations have challenged these dogmas. First, HCC could develop on normal liver, and predisposing genetic factor and genetic drivers involved in tumor initiation remain poorly described [53]. A simple clinical observation supports the fact that HCA is not a stochastic and isolated tumorgenic event: 40% of patients with HCA have multiple HCA in the liver suggesting an individual predisposition to develop this rare disease [34]. Also, several genetic disease and environmental factors favored hepatocytes proliferation and benign tumors initiation. Moreover, since several decades, the major HCA risk factors, oestrogen and androgen consumptions, have been identified as classical genotoxins [54–56]. Association between estrogen exposure and HCA occurrence was first described in the seventies when oral contraception was of widespread use in western countries [4, 55, 57]. In addition, tumor regressions after estrogen withdrawal have been reported [56]. It underlined that HCA is a hormonal-driven benign tumor. Nevertheless, estrogen exposure due to oral contraception is frequent, but HCA occurrence is rare (around 3/100,000) [55]. It seems that others genetic and/or environmental factors are required to promote HCA development. More recently, the use of a third generation of oral contraceptive with lower dose of estrogen could have modified the epidemiology of HCA [58]. However, robust epidemiological data comparing these two periods in western countries are lacking. In addition, the incidence of HCA in eastern countries, where oral contraception is not frequently used, remains to be evaluated. Differences in incidence and molecular subtypes of HCA between eastern and western countries could help to understand the role of estrogen exposure and other risk factors like obesity and alcohol consumption in the development of benign liver tumors [46, 59]. When we analyzed the spectrum of mutations of *HNF1A* in HCA, we also showed that *HNF1A* somatic mutations were frequently caused by G to T transversion suggesting a genotoxic exposure at the origin of the mutations [60]. Causes of this genotoxic signature remain to be elucidated, and the role of oestrogen exposition in this genotoxic damage should be further analyzed. A hypothesis is that HCA development could be favored by both a genetic predisposition in combination with an exposure to different genotoxic agents.

In this line, predisposing genetic factors like *HNF1A* germline mutation related to MODY3 diabetes and *GNAS* mosaic somatic mutations related to McCune Albright disease are strong risk factors of adenomas occurrence [19, 50]. Moreover, patients with glycogenosis type IA defined by germline inactivating mutation of glucose-6 phosphatase have a huge risk to develop multiple HCA during their followup [61–63]. All these data underlined that hepatocellular benign tumors are often developed on a predisposing abnormal liver background. This hypothesis could be called “benign tumorigenic field effect” as a mirror of the “carcinogenic field effect” described for HCC developed on cirrhosis. The “benign tumorigenic field effect” is a conjunction between genetic (*HNF1A *germline mutation, *GNAS* mosaic postzygotic mutation, and others unknown modifier genes) and environmental factors (oestrogen and androgen expositions) [20, 56, 57, 60, 64]. In addition, we showed a role of CYP1B1, a cytochrome p450 unit involved in detoxification of catechol estrogens, in the occurrence of HCA [25]. We identified a germline *CYP1B1*-inactivating mutation in 12.5% of patients developing *HNF1A*-inactivated HCA. Moreover, when analyzing the spectrum of somatic mutations in *HNF1A*, we identified a genotoxic signature typical of molecule inducing adduct to DNA at guanine [60]. Thus, a combined genetic predisposition and genotoxic effect could explain the frequent occurrence of multiple HCA in the same patient, and despite that the surrounding nontumor liver appears to be mainly “histologically (sub)normal,” the liver is tumorigenic. 

## 4. Conclusion

A long path has been walked in the area of hepatocellular benign tumors since Edmonson described the association between HCA and oral contraception [4]. Now, the discovery of genetic drivers of HCA has refined our knowledge of the life history of HCA from risk factors and clinical features to the risk of malignant transformation. However, several goals are still unmeet. First, the risk factors leading to HCA development are partially understood. Most of the patients have no known genetic factors predisposing to HCA occurrence. Moreover, all patients with genetic alterations predisposing to HCA will not develop tumors. So, additional genetics and environmental factors remain to be discovered. Thus, in addition to activating mutations of *β*-catenin, other genetic alterations leading to full malignant transformation have to be deciphered. Finally, several driver genes of benign tumorigenesis are still unknown, especially in the group of inflammatory HCA without known driver mutations and unclassified HCA. Ultimately, these genetic alterations will constitute therapeutic target for biotherapy that will be used in unresectable HCA or in other malignancies harboring the same genetic events.

## Figures and Tables

**Table 1:** Genotype/phenotype classification of hepatocellular adenomas.

Group	%	Genetic alteration		Pathway dysregulated	mRNA markers	Protein markers	Clinical association	Histological phenotype
*HNF1A*-mutated HCA	30–45%	*HNF1A *	Tumor suppressor gene	Activation of glycolysis, fatty acid synthesis, and mTor pathway	Decrease LFABP1 UGT2B7	lack of LFABP1 expression	Adenomatosis and association with MODY 3 diabetes (*HNF1A* germline mutation)	Diffuse steatosis

*CTNNB1*-mutated HCA*	10–15%	*CTNNB1 *	Oncogene	Activation of Wnt/catenin pathway	Increase GLUL LGR5	overexpression of glutamine synthase and nuclear *β*-catenin	Risk of malignant transformation Male	Cell atypia and cholestasis

Inflammatory HCA*	40–55%	*IL6ST* (65%)	Oncogene	Activation of JAK/STAT pathway (“oncogene-induced inflammation”)	Increase SAA CRP	SAA and CRP over-expression	Obesity and high alcohol intake Inflammatory syndrome	Inflammatory infiltrate Sinusoidal dilatation Dystrophic arteries
		*STAT3* (6%)						
		*GNAS* (5%)						
		Unknown (24%)					

Unclassified	10%	Unknown						

*50% of *CTNNB1*-mutated adenomas are also inflammatory.
